# Analysis of factors influencing public employees' work cognition under a public health crisis: A survey of China's response to COVID-19

**DOI:** 10.3389/fpubh.2022.938402

**Published:** 2022-08-19

**Authors:** Anhua Yang, Xue Zhou, Muhammad Tayyab Sohail, Muhammad Rizwanullah, Bo Dai

**Affiliations:** ^1^School of Public Administration, Xiangtan University, Xiangtan, China; ^2^School of Management, Hunan University of Technology and Business, Changsha, China

**Keywords:** public health crisis, public employees, work cognition, influencing factors, COVID-19

## Abstract

The work cognition of public employees lays importance on tackling an escalating health crisis situation. At the micro-level and macro-level, different factors contribute to different degrees of public employees' work cognition. However, there are limited studies examining the work cognition of public employees and its influencing factors, particularly in situations such as a public health crisis. Our research takes China's response to COVID-19 as an example. The data have been taken from six Chinese provinces, Hunan, Hubei, Jiangsu, Shanxi, Henan, and Shandong, through a total of 738 questionnaires and telephonic interviews. Furthermore, this study used a logistic multiple regression model to analyze the factors that influenced the public employees' work cognition when working under a public health crisis. The results of our study showed that at a micro-level, the educational background, attitudes, and actions (initiative, responsibility, administrative capacity, and timeliness of feedback), and their level of concern with work influenced the work cognition of the public employees. At a macro-level, we found that it was the anti-epidemic measures that most influenced public employees' work cognition. Our findings provide important policy implications for emergency preparedness and handling of major emergencies, and have important reference value for the management of public employees and the improvement of national governance capabilities under similar major challenges in the future.

## Introduction

The rapid and large-scale flow of people, commodities, and ideas across the world has become the driving force behind pandemics and leads to various kinds of world health crises (1, 2). The increasing frequency of public health emergencies, especially concerning new infectious diseases, has become a noteworthy issue because they pose a huge threat and loss to human life and are difficult to deal with. Consequently, the uncertainty and complexity of these public health crises have created new requirements for emergency management. In recent years, COVID-19 is spreading at a lethal pace and currently has been a major example of the new infectious diseases that can spread from human to human and lays a crucial threat to humanity. Governments face enormous challenges to cope with this new virus, adopt new policies, support vulnerable communities and individuals, make progress toward achieving sustainable development goals, and find new ways to achieve results while under great pressure and emergency (3, 4). COVID-19 variant of the omicron strain was first detected in South Africa on 9 November 2021. The overall global risk assessment for the Omicron strain is very high, according to the World Health Organization. In 2022, with the rapid spread of the more infectious omicron and the omicron subtype variant BA.2, the infection rate in Germany has broken records for 4 consecutive days, and the number of cases in Austria has reached a new high. Even China, the template for fighting the epidemic, has been breaking records for days, and many countries are at risk of a rebound. Consequently, this situation once again reminded mankind that coping with such a major public health crisis poses a long-term and arduous challenge.

However, the model adopted to fight against COVID-19 in China provides us with a unique sample of research. In December 2019, clusters of patients suffering from pneumonia that was coming from an unknown pathogen were reported in Wuhan, China (5). Unfortunately, COVID-19 then spread rapidly throughout the city and other cities in Hubei Province, and eventually spread to other provinces across the country. With the spread of the epidemic, a highly complex, intractable, and extremely uncertain public health event unfolded, which posed a serious threat to not only the lives of people in China but also to the population throughout the world. In order to deal with this challenge, China has made great efforts to carry out effective emergency response, including mobilizing the public service sector. For example, a large number of public employees have been moved to the front line of the fight against COVID-19, with many of these public employees even called back to work when on their way home for New Year's Eve. In addition, their Spring Festival holiday was immediately canceled in order to build a tight defense line against the epidemic. Thus, in this way, China has formed a Chinese model to fight against COVID-19 (6, 7). In China, a crisis response has never been taken to the height of “Overall War,” which indicates that the country's response to this epidemic has reached unprecedented heights. Two months after taking these measures, China had achieved a phased victory in its prevention and control of the epidemic, an achievement that is inseparable from the arduous efforts and sacrifices made by public employees.

Studies have shown that an employee's psychological cognition is the most critical factor affecting performance (8). Therefore, the work cognition of public employees has become an important factor in measuring the effectiveness of responses to public health crises. The level of work cognition directly affects the understanding and recognition of the work of public employees and in turn, their motivation and performance. The purpose of our research is to strengthen the management of public sector employees in a public health crisis and ensure the effective functioning of the system by examining the work cognition of public sector employees in this state. This greatly affects the way we respond to major public health crises and therefore the success of the response. In this study, we specifically try to respond to the following questions, as these questions have become important issues in need of urgent research:

How do public employees view their work when responding to public health crises?

Are there any differences in the cognition of different public employees regarding their work during a public health crisis?

What factors influence their work cognition?

What actions can we take?

Consequently, the current paper seeks to make the following contributions to the existing literature. First, it emphasizes the attention to the work cognition of public employees and the importance of the influencing factors. The past literature regarding crisis management generally ignores the concept of public employees and also lacks research on public employees' work cognition while working under high intensity or pressure during a public health crisis. The level of work cognition directly affects public employees' understanding of their work and thus influences their motivation, which consequently affects their work performance. As a result, the work cognition of Chinese public employees who have been strongly mobilized has become an important factor to measure the effectiveness of the response. Additionally, public employees' work cognition is directly related to policy effectiveness and administrative performance in emergency and crisis situations.

Moreover, the second contribution of our current study has been focusing on the micro-level of public management. Looking into past literature, several researchers have shown that outbreaks of COVID-19 may lead to a real mental health crisis, particularly in countries with a major challenge (9). However, the academic community has relatively more results in the study of COVID-19 from the perspective of public management focusing on the macro-level emergency management, and prevention and control measures (10, 11). The severe and complex situation urgently requires scholars to provide intellectual support from the micro-level of public management. Consequently, while managing public health crises, the public employees have been one of the most important players, working as either leaders or executors, and their work cognition is thus directly related to the response to public health events. Our paper adopts the regression analysis method to investigate the empirical case study on the response to COVID-19 in China, and explore the key factors influencing the work cognition of public employees. Furthermore, the second section of the study provides a theoretical basis for response to COVID-19 and work cognition, the third section describes the research method adopted for the study analysis and data collection, the fourth section presents results and discussion, the fifth section gives the conclusion, the sixth section details the implications, and the final section includes limitations.

## Theoretical basis

### Response to COVID-19

Since the outbreak of the COVID-19 epidemic, relevant academic research has been carried out to prioritize the matter of the epidemic. Currently, experts are mainly focusing on the areas of epidemiology (12, 13), treatment (14, 15), mental health (16, 17), prevention, and control (18, 19) of the COVID-19 epidemic. As early as 2020, the Chinese Center for Disease Control and Prevention conducted a preliminary investigation of the genome, virus isolation methods, specific cytopathic effects, and morphology of the COVID-19 (20). With the rapid spread of the virus all over the world, experts combined their knowledge with the clinical manifestations of COVID-19 to propose reference diagnosis, treatment (21, 22), prevention, and control plans (23, 24), including emergency management of hospital (25). Now that the COVID-19 epidemic poses a serious threat to human society across the globe, there is a great necessity for governments to act as risk communicators, emergency leaders, resource coordinators, and innovation enablers during this public health crisis (26). The success of a country's fight against COVID-19 depends on the national system (27), the mode of fighting COVID-19 (28), private actors (29), and the public (30). Therefore, more and more studies are studying the capabilities, experiences, and lessons of countries in fighting the epidemic from a more macro-level. For instance, to improve policy responses and the capacity of governments, the Singapore Government mainly overcame the panic of the masses through timely communication (31–33); Capano et al. (34) studied the capacity and incapacity across governments in their responses to natural experiments, such as COVID-19; Wang et al. (29) explained how the private sector can be a leader in initiating and managing public-private partnerships during a crisis, and how stakeholders in such collaborations can work together to effectively respond to the crisis. This does help to expand the scope of the practice and research of emergency management. However, the micro-unit of public service is also vital and deserves attention. Academic circles generally ignore the importance of public employees in the public service system in responding to public health events, particularly their psychological status. So, our current study focused on the work cognition of public employees during the COVID-19 epidemic. This is also the gap and weakness of current emergency management research.

### Work cognition

Cognition can be defined as “the mental action or process of acquiring knowledge and understanding through thought, experience, and the senses.” Work cognition involves the development of thought patterns or schema that have features, aspects, feelings, and ideas associated with past and present work experiences (35–37). In other words, work cognition is composed of an accumulation of mental descriptions and past judgments that then impact a given work experience in the present (38). In this article, the work cognition of public employees refers to the public employees' knowledge, understanding, and perception of their work, as well as their attitudes and views on it. In an effort to distinguish between what employees think about their work and what they feel about their work, Zigarmic et al. (39, 40) called for the separation of the cognitive and affective aspects of appraisals by making clear distinctions in the way they are conceptualized and measured. Later, Nimon et al. (41) developed the Work Cognition Inventory (WCI) to measure the various schema that are associated with the cognitive appraisal of the workplace. Although the psychometric scale of the former WCI was considered an exemplar by Newman et al. (42), the WCI does not measure concepts such as workload, task variety, performance expectations, and procedural fairness, which all play important roles in explaining how employees are engaged in work. Thus, Nimon et al. (43) presented a revised version of the Work Cognition Inventory (WCI-R) which mainly includes autonomy, collaboration, connectedness with colleagues, connectedness with the leader, distributive justice, procedural justice, feedback, growth, and meaningful work. However, these two WCIs measure the cognitive factors related to employees' passion for work, while other studies on work cognition mainly relate to employee happiness (44), satisfaction (45), and resignation (46). In fact, there are few studies on employees' work cognition and even fewer studies on the work cognition scale. Therefore, the existing research is still immature, and the depth of research on the influencing factors is not precise enough yet. Despite some useful research carried out by academics, work cognition is not incorporated into the cognitive structure of public employees. Furthermore, currently, only a few scholars have studied the cognition of public employees. An example is Xia (47), who divided the cognitive structure of public employees into three aspects: political cognition, professional cognition, and social-cultural cognition. However, the work cognition of public employees, particularly in situations such as a public health crisis, has not yet attracted the attention of researchers. In addition to political, professional, and socio-cultural cognition, we believe that work cognition is also crucial. This is because work cognition is not the exact equivalent of occupational cognition. In addition, as social mobilization is often needed to address significant public health crises, mobilization within the public service system is required from the first instance. In this context, therefore, the vast number of public employees experience virtually no changes to their duties except in the way they command, administrate, and execute their work, as in the case of fighting against COVID-19 in China. Therefore, in times like these, work cognition replaces occupational cognition. At this moment, work cognition is extremely important to the effectiveness of public services, but there are few studies in the academic community. Therefore, it is also an important motivation for our study.

## Materials and methods

The public employees mentioned in this article refer to the staff of the administrative organs, including the staff of the legislative and judicial administrative organs and their affiliated departments, and the members of political parties and social organizations supported by national public finances (48).

### Data collection

In this article, a quantitative research method is adopted. The data used in the study are obtained from the “Cognitive survey of public employees on the fight of the COVID-19,” which was organized by our research team. In order to respond to the call for “Home Isolation” during national epidemic prevention and control, and to ensure the timeliness and reference of the research work, the questionnaire survey uses an online survey method, combining two sampling methods of chance sampling and stratified random sampling. To improve the validity of the questionnaire and the relevance of the study, we surveyed public employees in six provinces across China and conducted a pre-survey by chance sampling in Xiangtan City, Hunan Province. After receiving feedback from the respondents of the pre-survey and following repeated amendments by experts, a formal questionnaire was designed. As the conditions and the prevention and control measures could vary during different periods of the outbreak, the survey was conducted in two phases, that is, March and August 2020. A total of 10 public employees were selected from the six provinces of Hunan, Hubei, Jiangsu, Shanxi, Henan, and Shandong who would then recruit volunteers for our questionnaire survey. Volunteers then distributed the questionnaire to public employees in their respective provinces by stratified sampling. In addition, we conducted telephone interviews that lasted over 30 min each with the above 10 public employees. A total of 802 questionnaires (pre-survey questionnaires are not included) were collected during the two survey stages. Due to the large content of our questionnaire, it takes at least 3 min for respondents to complete the questionnaire. It is impossible to complete the questionnaire if the online answer time is <1 min. According to the characteristics of the online questionnaire and the answering time of the questionnaire, 16 questionnaires with an answer time <60 s were eliminated, 13 questionnaires that had been repeatedly submitted by the same IP address were eliminated, and 35 questionnaires with incomplete information or failed to pass the logical consistency check (49, 50) were also eliminated. This meant that a total of 64 invalid questionnaires were eliminated, leaving 738 valid questionnaires, with an effective questionnaire rate of 92.0%. Based on the above method and the distribution of the valid samples, the data used and the results obtained in this article are highly credible.

### Questionnaire design

This article chooses China's response to the COVID-19 epidemic as its case study, refers to Nimon's Work Cognition Scale (41, 51) and the revised version of the Work Cognition Scale (43, 52), and then conducts a hypothetical analysis, at both the micro- and macro-levels, of the factors influencing public employees' work cognition on the response to a public health crisis. Based on previous research, we consider that the micro dimension may include individual characteristics, attitudes, actions, and levels of concern for their work, while the macro dimension mainly includes anti-epidemic measures. The questionnaire is therefore divided into four parts: the basic personal profiles of public employees, behavior and attitudes toward the response to COVID-19, the level of concern for the fight against COVID-19, and the evaluation of the measures taken for fighting against COVID-19.

#### Dependent variable

This article takes the level of public employees' work cognition in the prevention and control of the COVID-19 epidemic as the dependent variable. This dependent variable is then measured by “whether they value their participation in epidemic prevention and control.” The answer “Yes” is then assigned a value of “1,” and the answer “No” is assigned a value of “0.”

#### Independent variable

##### Individual characteristics

###### Gender

Public employees have different roles in their place of work and in their families due to gender differences. Typically, women take on relatively more family-related affairs and are able to spend less effort and time at work than men. Consequently, as the COVID-19 outbreak occurred during the Chinese Spring Festival holidays, more attention was required from women in taking care of family affairs. With women's attention focused on the family, the men's role had relatively more weight and stress with regard to the nationwide mobilization for the fighting against COVID-19. For this reason, men may have a better understanding of the work related to the prevention and control of the outbreak than women. It has also been found in a recent study that men are more realistic and objective oriented (53, 54).

###### Educational background

The more educated the public employees are, the better they are at predicting and tackling the developmental tendencies of the epidemic, and the better they are at understanding and perceiving the work related to epidemic prevention and control. Therefore, highly educated public employees have a clearer sense of the general picture of epidemic prevention and control. It is also found in the case of adopting online teaching modes during COVID-19 (55).

###### Post and rank

The higher their position, the more information they can obtain on the situation. Finally, armed with the most information possible, the more accurate the research and judgment of the epidemic situation can be, and thus the higher the level of cognition of the work on epidemic prevention and control.

###### Work experience

Public employees who have worked for several years are more experienced in coping with emergencies and are more sophisticated in their approach to epidemic prevention and control. Therefore, the deeper and more thorough the understanding of epidemic prevention and control, the higher the level of cognition in this subject.

##### Attitudes and actions

###### Initiative

The initiative of public employees is evaluated by asking the question “whether it is necessary to return to work early during the COVID-19 outbreak.” Psychology sees the initiative as a motivational manifestation of a person's behavior in a psychodynamic state. The more motivated the public employees are, the more intrinsic motivation they will devote to the prevention and control of the outbreak, and therefore the higher the level of cognition of epidemic prevention and control.

###### Sense of responsibility

This is measured by the responsibility that public employees have through asking “whether they are doing everything possible to complete each job.” The stronger the sense of responsibility, the more they regard the completion of tasks related to epidemic prevention and control as their duty. This, in turn, indicates a higher level of cognition of epidemic prevention and control. It is also observed in online communities that peers help each other when a sense of responsibility is established among users (56, 57).

###### Overall consciousness

The public employees' overall consciousness is measured by “whether they are willing to contribute when the country needs them.” The more public employees are aware of the big picture, and the more they are able to grasp it from a national perspective, the higher their cognition of epidemic prevention and control. Consciousness and awareness of threat due to the negative influence of certain objectives are significant factors behind human decision-making (42, 58).

###### Spirit of audacity

A public employee's spirit of audacity is evaluated by “whether they always require clear instructions from their superiors.” The more they dare to take responsibility, the more they will focus on how to take effective measures to carry out the work related to epidemic prevention and control. In this way, they will also stop being afraid of being held liable for incorrect decision-making or implementation. Hence, they will have a higher level of cognition of that work.

###### Administrative capacity

The administrative capacity of public employees is measured by “the leader thinks that you complete the work faster and better than other colleagues.” The stronger the administrative capacity of the public employees, the clearer their understanding will be of the content, division of labor, and implementation of the prevention and control measures, and the more rapid and efficient they will become in completing their tasks. Hence, they will have a higher level of cognition of the work related to epidemic prevention and control.

###### Timeliness of feedback

The timeliness of feedback on the work of public employees is measured by “how often do they report to their superiors.” In the fight against the epidemic, the more times feedback is given to their superiors, the more guidance they can receive from them, and the higher their cognition will become on the prevention and control of the outbreak.

##### Level of concern with work

In accordance with the Likert scale, the possible answers to the questions are as follows: “very concerned,” “relatively concerned,” “not concerned,” “not very concerned,” and “not concerned at all”.

###### Disclosure of epidemic information

Regarding how concerned public employees are with the disclosure of information, giving the public the right to know, reducing the panic caused by the outbreak, and helping the public make correct behavioral choices based on the information of the outbreak. This shows that the more aware they are of the prevention measures and control of the outbreak, the higher their level of cognition.

###### Propaganda and dissemination of information on prevention and control

The more public employees are concerned with the propaganda and the dissemination of information regarding the prevention and control of the epidemic, as well as raising public awareness and directing the public to behave in a correct protective manner, the higher level of cognition they have on the prevention and control of the outbreak.

###### Production and supply of medical supplies

During the outbreak, medical supplies are in high demand and resources are scarce for healthcare workers treating confirmed COVID-19 cases at the front line. The more public employees pay attention to the production and supply of medical materials, the more beneficial it is for the medical personnel to help patients overcome the virus. Thus, the public employee's attention to this indicates that they have a higher cognition of epidemic prevention and control.

###### Disinfection and epidemic prevention in public places

Public places provide a good place for the transmission of COVID-19. Effectively blocking the transmission of the virus is an important part of epidemic prevention and control. Therefore, the more public employees value the disinfection of public places and the more attention they pay to cut off the pathways of transmission of COVID-19, the higher level of cognition they have of epidemic prevention and control.

###### Rumor investigation and punishment

Because of the rapidity and anonymity of the spread of rumors, it is difficult to track down and manage them. In addition, rumors are harmful and can easily cause social instability. Therefore, the more public employees pay attention to the investigation of rumors, the punishment for spreading rumors, reassuring the public, and maintaining social stability, the more cognitive in epidemic prevention and control.

##### Anti-epidemic measures

###### Effectiveness

Since the outbreak of COVID-19, the Chinese government has continuously, and in due time, adjusted its epidemic prevention and control measures based on the changes in the general tendency of the outbreak. However, the effectiveness of the prevention and control measures is critical. The more effective the prevention and control measures are, the more accurately the public employees can grasp and perceive the work related to epidemic prevention and control, and thus, the higher their work cognition.

###### Deficiencies

At the outset of the outbreak of COVID-19, problems, such as unpreparedness for an emergency, incapacity for local government's response, inadequate sectoral coordination, and insufficient participation of social organizations, at one point led to the overwhelming of some local governments. These problems severely affected the prevention and control of the outbreak, as well as the response of public employees to it, which then further affected public employees' cognition of epidemic prevention and control.

## Results and discussion

### Descriptive analysis

#### Survey sample

As can be seen from Table 1, the ratio of male to female public employees in the sample is close to 1:1. The employees with age ranges of 21–30, 31–40, and 41–50 years were evenly distributed in three stages. In addition, the majority of the candidates had a bachelor's degree, accounting for 57.18% of the total sample, and those having a bachelor's degree and above constituted 78.32% of the total sample, indicating that the educational level of public employees was generally high. With regard to rank, division-level cadres and above accounted for 4.74%, section-level cadres accounted for 25.20%, senior clerks accounted for 38.86%, and junior clerks accounted for 31.17% of the total sample, indicating therefore that the rank of public employees in the sample was predominantly below the division level, and so they enjoy fewer chances to participate in the decision-making process of major issues. More than half of the sample of public employees had either <5 years of work experience or more than 20 years, while those with 6–20 years of work experience were distributed evenly. With regard to the survey on whether public employees value their participation in epidemic prevention and control, the statistical data show that 679 public employees answered “Yes,” accounting for 92% of the total sample size, and 59 public employees answered “No,” accounting for 8% of the total sample size. From the overall results of the sample, public employees have a high level of cognition regarding the prevention and control of COVID-19, but there are also a few public employees who have a relatively low level of cognition but are acceptable to participate in the study (36, 43). The basic demographic information of the sample indicates that the characteristics of the sample are similar to the actual situation of China's public employees, with strong representativeness and reference value.

**Table 1:** Survey sample.

**Variable**	**Classification indicator**	**Sample size/person**	**Percentage/%**
Gender	Male	391	52.98
	Female	347	47.02
Educational background	Junior high school and below	16	2.17
	Junior college	108	14.63
	Senior high school	36	4.88
	Undergraduate	422	57.18
	Postgraduate and above	156	21.14
Post and rank	Junior clerk	230	31.17
	Senior clerk	287	38.89
	Section level	186	25.20
	Division level and above	35	4.74
Work experience	1–5 years	246	33.33
	6–10 years	135	18.29
	11–15 years	86	11.60
	16–20 years	80	10.80
	20 years and above	191	25.80

#### Cognitive differences at work

##### Differences in post and rank

**Figure 1:**
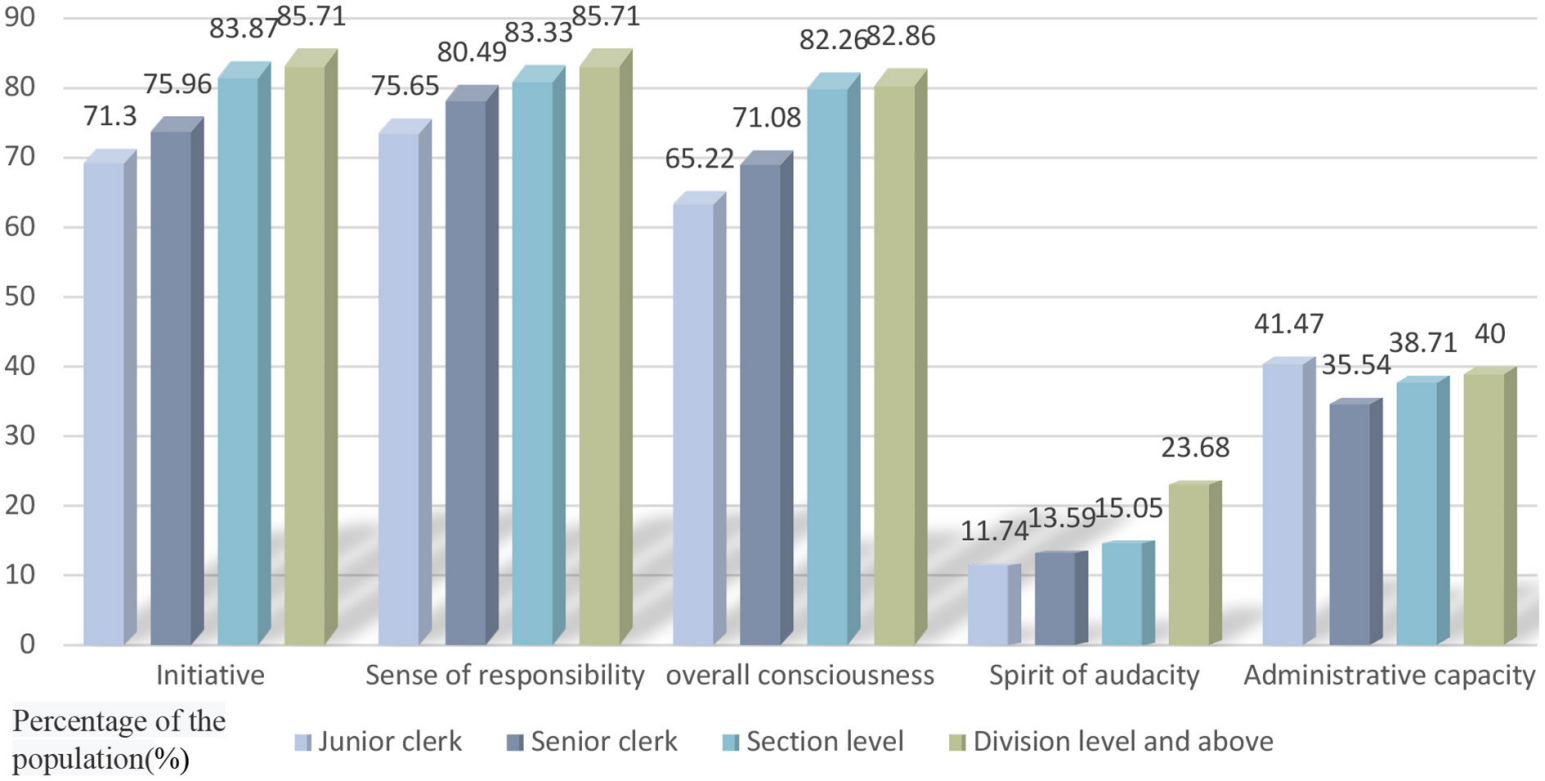
Differences in cognition of fighting against COVID-19 at different posts and ranks.

According to the analysis of the sample in Figure 1, obvious differences can be found in the cognition of public employees with different job levels. The overall trend shows that the higher the office the public employee holds, the more initiative they are in their work, the stronger administrative capacity and responsibility they have, the greater their spirit of audacity, and the greater their ability to see the big picture. However, of more interest is the finding that junior clerks show stronger administrative capacity than senior clerks and cadres working at section-level, department-level, and above. A possible explanation is that due to the upsurge of public employees in recent years, the competition during public employees' examinations in China is fierce. Thus, the public employees who are able to break through multiple assessments and enter the administrative system have relatively strong working abilities. In addition, junior clerks have enrolled as public employees more recently and are therefore still eager and confident in their careers, hence attaching greater importance to COVID-19 prevention and control. For them, an assignment from their leaders is worthy of great attention, and thus they tend to deliver work in a time-efficient manner and with as high quality as possible. This is the most prominent feature in the performance of the Chinese township cadres.

##### Differences with different work-experience levels

**Figure 2:**
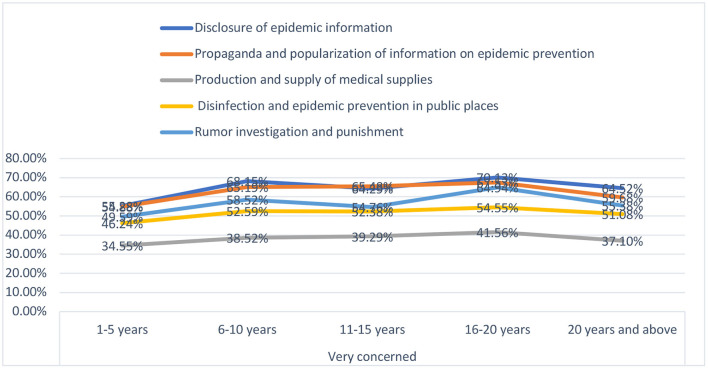
Differences in cognition of fighting against COVID-19 with different work-experience levels.

As shown in Figure 2, we find that public employees who have different career lengths show clear and particular differences in their work cognition. The overall trend shows that among public employees with a working life of <20 years, a higher proportion considers themselves “very concerned” about the prevention and control of COVID-19, but the proportion then decreases once they have worked for over 20 years. A possible explanation is that the longer the working period, the higher the level of public employees' cognition, but when the length of work reaches a certain stage, the public employees will begin to slack in their work, giving insufficient attention to the prevention and control of the outbreak, and having a relatively lower level of cognition.

### Multiple regression analysis

#### Model building

Our research focuses on exploring the influence of multiple independent variables on a dependent variable, where the influence of each independent variable on the dependent variable may be affected by other independent variables. That is, their actual influence may be different from their product-moment correlation coefficients. Therefore, multiple regression analysis is needed to solve the problem of using multiple independent variables to estimate or predict the value of a dependent variable and to make clear the actual influence of different independent variables on the dependent variable (59). The dependent variables in our study are dummy variables (dichotomous variables), and the independent variables are mostly dummy variables (categorical variables and ordinal variables). The value of public employees' work cognition level in fighting against COVID-19 is 0–1. The multiple regression model is expressed as follows:


(1) $$\begin{array}{l} p_{\text{i}}=F(y)=F(\beta_{\text{0}}+\sum_{\text{j}=1}^{n}\beta_{j}\alpha_{ij})\\ \;=1/\left\{1+\exp\left[-\left(\beta_{\text{0}}+\sum_{\text{j}=1}^{n}\beta_{j}\alpha_{ij}\right)\right]\right\} \end{array}$$

Taking the logarithm of the above expression (1) to get the logistic regression model, the expression is as follows:


(2) $$\begin{array}{c} \text{ln}\;\frac{p_{i}}{1-p_{i}}=\beta_{\text{0}}+\sum_{\text{j}=1}^{n}\beta_{j}\alpha_{\text{ij}}+\mu \end{array}$$

Among them, i (1, 2, 3, … n) represents the i-th variable, *p*_*i*_ represents the i-th public official's level of cognition regarding the prevention and control of COVID-19, β_0_ is the constant term of the regression Equation (2), β_*i*_ is the regression coefficient of the influencing factor, α_*ij*_ is the explanatory variable, indicating the j-th influencing factor of the i-th person, and μ is random error.

#### Multicollinearity diagnosis

In order to prevent the high correlation between the independent variables of the model from leading to the distortion of the estimated results of the model, a multicollinearity diagnosis of the selected independent variables is required before the logistic regression operation. In general, collinearity is present if Tolerance is < 0.1 or VIF is >10. Specifically, the value of Tolerance is between 0 and 1. Generally, when the Tolerance value is closer to 1, the collinearity among independent variables is weak, and the smaller value of Tolerance indicates that collinearity may exist. Tolerance < 0.1 indicates that multicollinearity is very serious. The value of VIF is between 0 and 10. Generally, the greater the VIF, the stronger the degree of multicollinearity among variables. As can be seen from Table 2, Tolerance values are all >0.1, and most variables are close to 1. The VIF values are all <10, and the values of all the variables are between 1 and 2.6, indicating that there is no multicollinearity problem among independent variables.

**Table 2:** Multicollinearity diagnosis.

**Independent variable**	**Collinearity statistics**	
	**Tolerance**	**VIF**
Gender	0.910	1.099
Educational background	0.704	1.420
Post and rank	0.694	1.441
Working experience	0.765	1.308
Whether it is necessary to return to work early	0.889	1.125
Doing everything possible to complete each job	0.554	1.805
The leader thinks that you complete the work faster and better than other colleagues	0.789	1.267
Willing to contribute when the country needs it	0.593	1.687
Requesting clear instructions from superiors regardless of it being an important or trivial matter	0.887	1.127
Frequency of reporting to the superior	0.939	1.065
Disclosure of epidemic information	0.538	1.859
Propaganda and popularization of epidemic prevention knowledge	0.426	2.348
Production and supply of medical supplies	0.532	1.879
Disinfection and epidemic prevention in public places	0.395	2.533
Rumor investigation and punishment	0.465	2.152
Effectiveness of epidemic prevention and control measures	0.593	1.686
Whether the emergency plan is sound or not	0.916	1.091
Local government emergency capacity	0.829	1.207
Coordination of various departments	0.829	1.207
Whether social organizations are fully involved	0.896	1.116

VIF is the abbreviation of variance inflation factor.

#### Logistic model estimation results

We used Stata15.0 statistical software to perform logistic regression on the questionnaire data, and the model regression results are shown in Table 3. There are generally three ways to measure how well a binary model (non-linear) fits: one is Pseudo *R*^2^, the second is the percentage of correct predictions, and the third is the likelihood ratio test statistics (60). The model estimation results in this article show that the Pseudo *R*^2^ is 0.387, the model prediction accuracy is 93.63%, and the log-likelihood value is −125.975, so the model can be considered a good fit.

**Table 3:** Logistic model estimation results.

**Type**	**Independent variable**	**B**	**S.E**.	**Wald**	**Sig**.	**Exp(B)**
Individual characteristics	Gender	−0.139	0.348	0.157	0.692	0.871
	Educational background	0.719***	0.269	7.146	0.008	0.487
	Post and rank	0.114	0.244	0.217	0.642	1.120
	Working experience	0.020	0.124	0.025	0.874	1.020
Attitudes and actions	Whether it is necessary to return to work early	1.063***	0.350	9.231	0.002	2.896
	Doing everything possible to complete each job	0.978***	0.320	9.319	0.002	2.659
	The leader thinks that you complete the work faster and better than other colleagues	1.383***	0.233	35.214	0.000	3.989
	Willing to contribute when the country needs it	0.423	0.390	1.172	0.279	1.526
	Requesting clear instructions from superiors regardless of it being an important or trivial matter	−0.271	0.173	2.460	0.117	0.762
	Frequency of reporting to the superior	−0.392*	0.204	3.679	0.055	0.676
Level of concern with work	Disclosure of epidemic information	0.879***	0.333	6.955	0.008	0.415
	Propaganda and popularization of epidemic prevention knowledge	0.058	0.363	0.026	0.873	1.060
	Production and supply of medical supplies	0.505*	0.284	3.166	0.075	1.657
	Disinfection and epidemic prevention in public places	−0.436	0.389	1.261	0.261	0.646
	Rumor investigation and punishment	0.696**	0.347	4.015	0.045	2.006
Anti-Epidemic measures	Effectiveness of epidemic prevention and control measures	0.795**	0.337	5.572	0.018	2.215
	Whether the emergency plan is sound or not	0.656*	0.386	2.883	0.089	1.927
	Local government emergency capacity	−0.617	0.465	1.758	0.185	0.540
	Coordination of various departments	0.254	0.406	0.391	0.532	1.289
	Whether social organizations are fully involved	−0.090	0.391	0.053	0.818	0.914
	Constant	−8.568***	2.421	12.524	0.000	0.000
	*PseudoR* ^2^		0.387			
	Correctly classified		93.63%			
	Log-Likelihood		−125.975			

* , ^**^, and ^***^ represent significance levels of 10, 5, and 1%, respectively. S. E., standard error; Sig., significance; Exp., Exponential. R^2^ indicates coefficient of determination.

The results of the above regression analysis are as follows:

The influence of individual characteristics

Among the individual characteristics, educational level is the main factor affecting public employees' work cognition. It can be seen from Table 3 that educational level is significant at the level of 1%, and it is positively correlated with the work cognition of public employees. This is consistent with expectations, indicating that the higher the level of education, the higher the level of knowledge, and the clearer the awareness and judgment of the situation, the stronger the ability to receive and understand the prevention and control measures of the epidemic, and hence the higher the cognitive level. However, the gender, the rank of the officer, and the length of time in work are not significant, which may be linked to the serious situation of the COVID-19 epidemic and the relatively high overall cognition of public employees. But the influence trend is generally consistent with the expectation.

The influence of attitudes and actions

In terms of attitudes and actions, the evaluation indicators, such as whether it is necessary to return to work early, making every effort to complete each task, completing tasks faster and better than other colleagues, and the frequency of job reporting, have a significant impact on public employees' work cognition.

- The question “whether it is necessary to return to work early” mainly measures the impact of an employee's initiative on work cognition. The significance level for this reaches 0.002, which is 1%, indicating that the initiative has a remarkable impact on the work cognition of epidemic prevention and control. As for the question on “doing their best to complete each task,” it mainly measures the impact of the sense of responsibility on work cognition. The more active public employees are in their work, the more active and engaged they are in the fight against the epidemic, and the higher their awareness of the fight against the epidemic. The significance level for this is 0.002, which is significant at the 1% level. The possible explanation is that the more responsible public employees are, the more they are familiar with and understand their work, and thus have a higher level of awareness of the work of fighting the epidemic. The significance level for this is 0.000, indicating that in work cognition, administrative capacity is the top priority among all influencing factors. Administrative capacity determines the understanding of the anti-epidemic work, the arrangement and implementation of plans, etc. The stronger the administrative capacity, the more thorough the understanding of the anti-epidemic work, and the higher the level of work awareness. The frequency with which an employee reports their work to their superiors mainly measures the timeliness of the feedback from public employees, which has a negative correlation with the work recognition of public employees, which is in line with expectations. A possible explanation is that the higher the frequency in which a public employee reports their work, the more time there is for feedback on the work, which is convenient for obtaining further guidance and help from their superior, and thus helps improve work cognition. The lower the reporting frequency, the more passive and negligent the government, which is not conducive to public employees' understanding of the anti-epidemic work.
- The influence of the “willingness to contribute when the country needs it” and “requiring clear instructions from the superior regardless of it being an important or trivial matter” on work cognition is not significant. A possible reason for this is that in the survey data, regarding the answer to the question “willing to contribute when the country needs it,” 98.78% of the public employees replied with “very much agreed” or “agreed.” This shows that Chinese public employees generally have good ability in terms of overall consciousness when it comes to the prevention and control of epidemics, so the impact is not obvious. The variable “requiring clear instructions from the superior regardless of it being an important or trivial matter” is not significant, but it is very close to the 10% significance level, which shows that spirit of audacity also has a certain influence on work cognition, but it is not the main influencing factor.

The influence of concern with work

In terms of the level of concern with work, the main factors that affect the public employees' work cognition are the disclosure of epidemic information, the production and supply of medical materials, and rumor investigation and punishment.

- Of these, epidemic information disclosure has the greatest impact, at a level of 1%. It means that the level of epidemic information disclosure is an important basis for decision-making and enforcement by public employees. During the outbreak of COVID-19, the untimely disclosure of information in the earlier period led to the massive spread of the virus throughout the country, creating numerous obstacles and difficulties in the process and greatly increasing the difficulty to control the outbreak. Later, when the disclosure of the epidemic information became transparent, information, such as the number of confirmed cases of COVID-19, the characteristics of human-to-human transmission, and hazards, were updated in a timely and accurate manner and on a daily basis. This largely eliminated panic, thereby further raising the level of public employees' work cognition (54, 55).
- The production and supply of medical materials are significant at the level of 5%, which is positively correlated with the dependent variable. A possible explanation is that the more public employees pay attention to the production and supply of medical materials, the more guaranteed the supply of medical materials is, the less panic the public employees feel about the virus, and the more confident they are in the prevention and control of the epidemic. Thus, the higher the level of work cognition becomes in the fight against the epidemic.
- Rumor investigation and punishment have a significant influence on epidemic prevention and control. A possible explanation is that the more public employees are concerned about investigating and dispelling rumors, the more attention will be paid to the prevention of the epidemic, and the more transparent and accurate the epidemic information will become. In addition, investigating rumors and punishing those propagating those rumors is conducive to eliminating public panic (56, 57).

The influence of anti-epidemic measures

Both the effectiveness of anti-epidemic measures and the perfection of emergency plans have a significant impact on public employees' work cognition.

- The effectiveness of the epidemic prevention and control measures is significant at the level of 5%, which is also positively correlated with the work cognition of public employees. Moreover, it is positively correlated with the job cognition of public employees, which is consistent with the expected direction. The possible explanation is that the effectiveness of anti-epidemic measures directly affects public employees' overall understanding of the prevention and control work and the implementation effect of the prevention and control measures. The more effective these measures are, the higher the level of public employees' work cognition. In addition, the effectiveness of the emergency plan also has a relatively significant impact on the work cognition of public employees. For this, a possible explanation is that the more imperfect the contingency plan is, the more disadvantaged the public employees are when making a rapid prejudgment of the general trend and responding in the event of an outbreak (56, 58).
- The three variables of local government emergency response capacity, coordination among departments, and participation of social organizations did not have a significant impact on public employees' cognition of anti-epidemic work. The possible explanation is that only public employees in a few provinces reported that local government emergency response capacity was insufficient, and the sample data were relatively small, so the impact on dependent variables was not significant. Linkage and social organizations to participate in all departments to coordinate effect is not obvious reason may be that the outbreak, the regional various departments to coordinate linkage, active participation in the social from all walks of life, ordinary people all isolation in the home, the nation together outbreak win the phases of the epidemic prevention and control, department of public employees to coordinate linkage are more satisfied, and social organizations to participate in the two variables of the samples data differences are not significant, so the impact on job cognition is not significant.

## Conclusion

It has been worthwhile to study public employee management because public employees have been the acting subjects of the government's decisions during this public health crisis. Furthermore, the work cognition of public employees is related to the administrative performance with which they deal with the public health crisis. However, the work cognition of public employees has not attracted sufficient attention from the academic community. Consequently, the current study uses questionnaire data from the early stages of China's COVID-19 crisis to explore the work cognition of public employees on epidemic prevention and control during a public health crisis, and then uses the logistic multiple regression model to explore its influencing factors.

First, in accordance with the results, the Chinese public employees generally have a high level of cognition of epidemic prevention and control. This shows that in response to such a major public health crisis as COVID-19, it is necessary and effective to adopt the Chinese model based on strong national mobilization, including unremitting efforts from all public employees. These were important factors in how China was able to effectively control the epidemic in a relatively short period of time. Second, different public employees have differences in their cognition regarding their work during a public health crisis. Different job levels and work experience can lead to different job perceptions. It is interesting to note that junior clerks show stronger administrative capacity than senior clerks and cadres working at section-level, department-level, and above. The longer the working period, the higher the level of public employees' cognition. But when the length of work reaches a certain stage, the public employees will begin to slack in their work, giving insufficient attention to the prevention and control of the outbreak, and having a relatively lower level of cognition. What is more, from a micro perspective, the work cognition of public employees is influenced by their educational background, attitudes, and actions (initiative, sense of responsibility, administrative capacity, and timeliness of feedback) and their level of concern with work (dissemination of epidemic information, production and supply of medical supplies, rumor investigation, and punishment). At the macro-level, it is significantly affected by the anti-epidemic measures.

## Implications

Our findings have important implications for governments to effectively organize and manage public employees and successfully respond to public health crises. First of all, there should be a focus on raising the educational level of public employees and laying a solid foundation for public health crises. An important finding of this study is that education is the most important factor among the personal characteristics affecting public employees' work cognition. This, therefore, requires a focus on academic qualifications during the selection of public employees and further strengthening of institutionalized and normalized learning mechanisms. Additionally, there should be increased emergency response capacity training, including leadership crisis training for cadres at higher levels and emergency management competency training for the majority of public employees. On the one hand, it lays a solid foundation for dealing with acute major challenges, and at the same time, it lays a good talent reserve for dealing with potential public health crises in the future.

Second, there should be a disclosure of information to improve the work recognition of public employees when under a public health crisis. Whether or not they pay attention to the disclosure of epidemic information is a key factor affecting the work cognition of public employees. Timely and accurate disclosure of information demonstrates the firm determination and confidence of the state in response to the incident, thereby enhancing the confidence of all public employees and so keeps them closely aligned with the national strategy. At the same time, it also provides them with a timely understanding of the situation and the progress of their work. Since the SARS incident, China has established a sound system of laws and regulations to respond to emergencies and has issued a series of acts regarding the disclosure of information. The “principle of openness and transparency” is clearly stipulated in these laws and regulations, and as a scientific method that has been concluded at the cost of numerous lives (61), requires strict compliance and implementation.

Third, a guarantee and incentive mechanism must be established to stimulate the motivation of public employees to work during uncertainty and crisis. In order to achieve high efficiency, it is often necessary to take unconventional measures, including strong accountability, so that the epidemic is the order and prevention and control is the responsibility, which has become the general program of the epidemic prevention and control work in China. However, in addition to punishment, effective incentives are crucial to arousing the enthusiasm of public employees, and an important strategy to maintain high enthusiasm is to improve their work awareness level. Responding to a public health crisis urgently requires strict management and assurance of incentives. It not only requires high standards (including accountability), but also requires political incentives, guarantees of treatment, and fault tolerance within a certain range, to enhance the sense of honor, belonging, and acquisition of public employees, further improving their job awareness. For example, rapid promotion is given to those public employees with a prominent performance so as to mobilize and stimulate their motivation, initiative, and creativity, and further enhance their responsibility and administrative capacity.

Finally, the development of emergency management systems and capacity for modernization must be strengthened and the ability to prevent major risks and address public health crises enhanced. The high level of work cognition of Chinese public employees has become an important reason for the effective control of the epidemic in China, which shows the necessity and effectiveness of the Chinese model to deal with the epidemic. However, the cost of this model is often huge. On the one hand, the high level of work cognition among Chinese public employees has become an important reason for the effective control of the epidemic in China, which shows the necessity and effectiveness of the Chinese model to deal with the epidemic, but on the other hand, the costs are enormous. In the face of a major public health crisis like COVID-19, not a single department or sector has the ability to deal with the situation alone (62). The effectiveness of the model is therefore based on a hyper-intense work environment and enormous psychological stress on all public employees. As the duration extends, the level of work cognition of public employees may continuously decrease and consequently its effectiveness. Therefore, we should take this epidemic as an opportunity to comprehensively summarize the deficiencies of China's emergency management system. Further improvements are urgently needed in the concept itself, as well as in the system, mechanism, talent, and resource protection. In addition, in order to advance the modernization of the national emergency management system and increase its capacity, weaknesses must be strengthened, loopholes closed, and blind spots exposed and solved.

## Study limitations

It must be noted that, as there is no standard scale for reference, indicators are mainly selected from the existing literature and management practice. The design of the scale is not comprehensive and accurate enough, and the setting of indicators can be further optimized in the future. What is more important is that public health crises are diverse, and different types of public health crises require different approaches. The research in this article is based on the major public health event, COVID-19, and it is mainly suitable for the public health crisis in the Chinese context and does not therefore necessarily apply to other countries. However, our research and findings on the work cognition of public employees as the acting subjects during public health crises will undoubtedly help to further develop research in this field and provide a theoretical basis and guidelines for the effective management of public employees when under public health crises.

## Data availability statement

The raw data supporting the conclusions of this article will be made available by the authors, without undue reservation.

## Author contributions

AY and XZ: conceptualization, investigation, validation, formal analysis, writing-original draft preparation, visualization, supervision, project administration, and funding acquisition. XZ: methodology, resources, and data curation. AY, XZ, and MR: investigation. AY, XZ, BD, and MS: reviewing and editing. All authors have read and agreed to the published version of the manuscript.

## Funding

Funding was received from Key Project of Education Department of Hunan Province of China (the construction of emergency management system and capacity-Soft Power-in Hunan Province Grant No. 20A492) and the National Social Science Fund of China (the impact mechanism and emergency response mechanism of agricultural enterprise performance under major public health emergencies due to high volatility of supply and demand Grant No. 72063008).

## Conflict of interest

The authors declare that the research was conducted in the absence of any commercial or financial relationships that could be construed as a potential conflict of interest.

## Publisher's note

All claims expressed in this article are solely those of the authors and do not necessarily represent those of their affiliated organizations, or those of the publisher, the editors and the reviewers. Any product that may be evaluated in this article, or claim that may be made by its manufacturer, is not guaranteed or endorsed by the publisher.
